# The association of right coronary artery conus branch size and course with ST segment elevation of right precordial leads and clinical outcome of acute anterior myocardial infarction

**DOI:** 10.15171/jcvtr.2017.07

**Published:** 2017-03-18

**Authors:** Samad Ghaffari, Mohammadreza Taban Sadeghi, Mohammad Hossein Sayyadi

**Affiliations:** Cardiovascular Research Center, Madani Heart Hospital, Tabriz University of Medicine, Tabriz, Iran

**Keywords:** ST Elevation, Precordial Leads, Anterior MI, Conus Branch, Right Coronary Artery

## Abstract

***Introduction:*** Coronary artery disease is the leading cause of death worldwide and electrocardiogram (ECG) is a reliable diagnostic tool to determine a myocardial infarction. The present study tried to compare the relationship between the ECG findings and angiographic findings in patients with acute anterior myocardial infarction.

***Methods:*** Seventy-four patients with acute anterior ST elevation myocardial infarction (Ant- STEMI) presenting to the emergency room in the first 12 hours after the onset of symptoms were studied. Upon admission, a full 14-lead ECG (including leads V3R and V4R) were performed. Angiographic and ECG findings, as well as clinical outcome were compared between two groups. The statistical tests including Chi-square and independent t-test were used for data analysis.

***Results:*** Small conus branch was seen in 52 (70.3%) and large conus in 22 ( 29.7%) patients. STE in right-sided leads and heart failure were significantly higher in small conus branch group versus large conus branch (88.6% vs 11.4%, *P* < 0.001 and 34.6% vs 9.1%, *P* = 0.02 respectively). There was no significant difference in mortality rate between the two groups (5.8% in small conous group vs 0% in large conus group, *P* = 0.55). There was a significant difference in major adverse cardiac events (MACE) between the two groups (51.9% in small conous group vs 18.2% in large conus group, *P* = 0.01).

***Conclusion:*** In patients with anterior MI, small conus branch was associated with higher rate of major adverse cardiac events mostly because of increased rate of acute heart failure.

## Introduction

Coronary artery disease is the leading cause of death worldwide and electrocardiogram (ECG) is a reliable diagnostic tool to determine a myocardial infarction. STE in lead V4R can indicate right ventricular infarction in patients with inferior infarction, which is associated with a poor prognosis and can lead to arrhythmias and conduction disorders and VF.^1-3^ Limited data indicated that STE in lead V4R in patients with anterior infarction is associated with a poor prognosis.^4^

Elevation of ST-segment in leads V3R and V4R has also been reported as an occasional finding in some patients with anterior ST elevation myocardial infarction (Ant-STEMI. However, there is a conflict on how these changes affect patients’ prognosis.^5-9^

The conus branch of the right coronary artery (RCA) often supplies blood to the right ventricular outflow tract. Previous studies have reported that specific ECG changes (elevation in the ST-segment in the precordial leads) appear in the event of a blockage or narrowing of the conus branch.^10-14^ STE in lead V1 accompanies the STE in lead V3R and indicates the presence of a small conus branch that does not reach interventricular septum. A dual circulation in the right paraseptal area of the interventricular septum protects against ischemia and prevents the right ventricle steal during ischemia and minimizes the damage caused by left ventricular ischemia during anterior infarction.^15^ Anatomic variations of this branch can be an important limiting factor leading to different conclusions about the frequency of arrhythmias, heart failure, and LVEF in different studies. ^16^

The basal portion of the interventricular septum is supplied with blood by septal branches of LAD, alone or in combination with the conus branch. The presence of a large conus branch may protect the right side of the interventricular septum which manifests with the lack of STE in lead V1. If a small conus branch is present, blood is supplied to both sides of the interventricular septum by LAD artery branches, hence interventricular septum is left unprotected during LAD occlusion which is indicated with STE in lead V1.^15^

Researchers also examined the relationship between RCA conus branch and pattern of STE in lead V1 and V3R during anterior MI. The patients were divided into two groups based on the STE amount in V1. There was a significant difference between the two groups in terms of STE in V3R and presence of small conus branch.^16^

Another study found that STE in aVR, V1, and V3R leads is associated with a small conus that had not reached the interventricular septum. The study examined the relationship between V3R, V1 and aVR during anterior STEMI and the main lesion in LAD and the nature of RCA conus branch. For this purpose, 142 patients with the first MI were enrolled. STE in aVR, V1 and V3R in cases with proximal occlusion to the first septal perforator was more than cases with distal occlusion. Out of the 60 subjects with proximal occlusion, 20 patients had a small conus branch (18 patients with STE in aVR and 15 patients with STE in V1) and 24 patients had a large conus branch (all patients had STE in aVR). The study revealed that V3R and aVR had a sensitivity of 84% and 90% and specificity of 66.7% and 64.9% in the detection of a small conus branch.^17^

Given the important role of the intraventricular septum in patients, there is a need for an appropriate strategy selected for patients presenting with an acute anterior myocardial infarction in order to prevent undesirable consequences. The intraventricular septum can have a double blood supply or a single blood supply from a branch of the LAD artery or conus branch, therefore, the present study tried to compare the relationship between the ECG findings and angiographic findings in patients with acute anterior myocardial infarction.

## Materials and Methods

### Methods

Patients with acute Ant STEMI presenting to the emergency room of Shahid Madani Hospital in the first 12 hours after the onset of symptoms were studied. Patients with devices in their heart, a history of previous myocardial infarction or heart failure, bundle branch block, or chronic renal failure were excluded. Samples were selected by convenient census sampling. All required information was obtained from medical and hospital records of the patients. Written consent was obtained for inclusion in the study from patients’ accompaniment and the study was approved by the Tabriz University Ethics Committee under the number 94/3-9/6. Upon admission, a full 14-lead ECG (including leads V3R and V4R) with a 25 mm/s speed and 1 mV/10 mm standard were performed. Coronary angiography was performed within 48 hours after admission. Coronary angiography was performed for four left coronary system views (RAO, LAO, AP cranial, AP caudal) and two right coronary system views (LAO, RAO). The lesion site in LAD artery in relation to the S1 branch and the conus branch type in right coronary artery were identified: small conus branch does not reach the IVS less than 0.5 mm and large conus branch reaches IVS greater than 0.5 mm.

The first sample of venous blood was taken within the first hour of patient hospitalization to assess cardiac enzymes and for 2 times every 8 hours repeated to assess the peak levels of enzymes.

Clinical heart failure was defined as congestion manifested in chest radiography or presence of bilateral crackles in lung auscultation.

Angiographic and ECG findings, clinical heart failure, cardiogenic shock, acute MR, mechanical complications including ventricular septal defect (VSD) and recurrent myocardial infarction (re MI), electrical complications (VF and VT in the first day of admission, LBBB, RBBB, AF, AV block), left and right ventricular LVEF, and mortality were examined. Finally, 74 patients entered the study, the relationship between STE in the right leads and conus size in predicting electrical and mechanical complications, mortality, and ventricular dysfunction was assessed.

### Statistical Analysis

All statistical analyses were performed in SPSS software (Version 17.0, SPSS Inc., Chicago, IL, USA) using descriptive methods (frequency, percentage, mean± SD). Normality of the quantitative data was confirmed using the K–S test. Chi-square test (or Fisher’s exact test if needed) were used to compare qualitative results and independent *t* test was used to compare quantitative variables. The *P* < 0.05 value was considered significant.

## Results

The mean age of the patients was 57.95±12.36 years (median 58 years). The youngest and oldest patients were 32 and 92, respectively. A total of 64 patients (86.5%) were male and 10 patients (13.5%) were female. Positive family history was observed in 3 cases (4.1%), hyperlipidemia in 9 cases (12.2%), hypertension in 25 cases (33.8%), diabetes in 13 cases (17.6%) and smoking in 36 cases (48.6%). The mean interval from onset of pain to the first ECG was 3.43±1.44 hours (median 1 hour). Minimum and maximum durations were 30 minutes and 6 hours, respectively.

The infarction site was anteroseptal in 28 cases (37.8%), anterior in 17 cases (23%), and anterolateral in 29 cases (39.2%). Mean STE in V3R and V4R was 1.45±0.73 mm and 0.89±0.36 mm, respectively. Mean STE in aVR and V1 was 0.84±0.24 mm and 1.38±0.76 mm, respectively. Mean total STE in the first ECG was 15.54±8.40 mm. Totally, STE in V3R or V4R was observed in 44 cases (59.5%).

Electric complications were observed in 10 cases (13.5%), including VF or VT in the first day in 7 cases (9.5%), VF or VT after the first day, AF and first-degree AV block, each in one case (1.4%).

Among mechanical complications, only heart failure was observed in 20 cases (27%).

The mean hospital stay was 6.36±4.51 days with a median of 5 days. The minimum and maximum lengths of stay were 2 and 35 days, respectively.

In cases with arterial involvement detected in angiography, one-vessel disease was observed in 40 cases (54.1%), and two- and three-vessel disease in 17 cases (23%). LML involvement of over 50% was observed in three cases (4.1%). The lesion site on LAD was proximal in all cases. LCX lesions of over 50% on LCX were observed in 27 cases (36.5%), and on RCA in 24 cases (32.4%). There were 53 cases (73%) of right dominance, 16 cases (21.6%) of left dominance, and 4 cases (5.4%) co-dominance. Small conus was observed in 52 cases (70.3%) and large conus in 22 cases (29.7%).

Table 1 shows the basic findings of cases with large and small conus branches. There was no significant statistical difference between the two groups. Only the amount of STE in V4R was significantly higher in the small conus group.

Totally, electrical complications were observed in 6 patients in the group with small conus branch, and in 4 patients in the group with large conus branch. There was no statistically significant difference between the two groups (*P* = 0.44).

Heart failure was observed in 18 patients in the group with small conus branches, and in 2 cases in the group with large conus branches.

There was a statistically significant difference (*P* = 0.02). Table 2 compares various electrical complications and mechanical complications in the two groups.

In-hospital death was observed in two cases (3.8%) and death during follow-ups was reported in one case (2%) only in the small conus branch group. No cases of death were observed in the large conus branch group. There was no significant difference in this respect between the two groups (*P* = 0.9). No significant difference was observed between small and large conus branch groups in terms of overall death rate (3 cases (5.8%) Vs 0 cases, *P* = 0.55).

The mean length of hospital stay of patients with large and small conus branch were 6.53±4.99 and 6.86±3.15 days, respectively. There was no significant difference (*P* = 0.77).

## Discussion

Acute right ventricular MI was observed in almost half of the cases with inferior MI and 40% of the cases with anterior MI.^17^ The right ventricular infarction is an independent risk factor for increased mortality in patients with acute STEMI who were treated by PCI.^18,19^ Although the majority of outcomes in patients with right heart complications were examined in the inferior infarction, its role is also significant in the anterior infarction.

The present study evaluated the relationship of RCA conus branch with STE in precordial leads in patients with acute anterior infarction. It was observed that small conus branch had significantly higher amounts of STE in the right leads and subsequently was associated with more heart failure compared to the large conus branch. STE in the right leads was also associated with higher rates of heart failure.

In a similar study, Ben-Gal et al observed that in patients with anterior MI, STE was significantly more in the right leads in the small conus branch than in the large conus branch.^15^ Another study by Zhong-Qun et al found that STE in aVR, V1, and V3R leads is associated with a small conus that has not reached the interventricular septum.^17^ However, no information was available in these studies about the outcome and incidence of heart failure.

The present study also showed that although not significant, the presence of small conus branch was associated with less electrical complications, lower LVEF, maximum CTnI and CKMB, frequent 3VD, and more death compared to large conus branch.

There are some reports about the protective role of conus branch of the right coronary artery in the intraventricular septum and STE in lead V1 or right precordial leads. Anatomic variations of this branch can be an important limiting factor leading to different conclusions about the frequency of arrhythmias, heart failure, and LVEF in different studies.

Lower number of enrolled patients and lack of long term follow up were major limitations of our study and larger studies with long term follow up are needed for detailed evaluation of this issue.

**Table 1:** Basic findings of cases with large and small conus branches

	**Small conus** **n=52 (70.3%)٭**	**Large conus** **n=22 (29.7%)٭**	**P value**
Age (year)	59.37±11.94^†^	54.68±12.97^†^	0.13^‡^
Gender			
Male	45 (86.5%)	19 (86.4%)	0.98^¶^
Female	7 (13.6%)	3 (13.6%)	
Family history	3 (5.8%)	0	0.55^¶^
Hyperlipidemia	5 (9.6%)	4 (18.2%)	0.3^¶^
Hypertension	16 (30.8%)	9 (40.8%)	0.39^¶^
Diabetes	8 (15.4%)	5 (22.7%)	0.44^¶^
Smoking^§^	26 (50%)	10 (45.5%)	0.72^¶^
Time from onset of pain (h)	3.62±1.42^†^	3.00±1.43^†^	0.9^‡^
STE in V3R>1 mm	13 (25%)	7 (31.8%)	0.54^¶^
STE in V4R>1 mm	3 (5.8%)	0	0.55^¶^
STE in V3R or V4R>1 mm	13 (25%)	7 (31.8%)	0.54^¶^
STE in aVR	0.78±0.25^†^	0.88±0.22^†^	0.19^‡^
STE in V1	1.42±0.75^†^	1.10±0.39^†^	0.1^‡^
Total STE	16.57±8.21^†^	13.09±8.53^†^	0.1^‡^
Infarction location			0.39^¶^
Anteroseptal	18 (34.6%)	10 (45.5%)	
Broad anterior	11 (21.2%)	6 (27.3%)	
Anterolateral	23 (44.2%)	6 (27.3%)	
CPK	838.88±87.66^†^	702.20±51.49^†^	0.24^‡^
First CTnI	0.26±0.24^†^	0.22±0.20^†^	0.45^‡^
First CKMB	45.10±8.90^†^	45.27±10.50^†^	0.96^‡^
Peak CTnI	81.31±11.67^†^	51.18±11.17^†^	0.43^‡^
Peak CKMB	103.96(0-360.01)^†^	552±201.16^†^	0.45^‡^

‏٭Number (percent). † Mean (Standard deviation). ‡ Independent sample *t* test ¶ Chi-square. § smoking the last three years.

**Table 2:** In hospital complications of MI among cases with large and small conus branches

	**Small conus** n=52 (70.3%)*	**Large conus** n=22 (29.7%)*	***P*** ** value** ^†^
VF or VT in the first day	5 (9.6%)	2 (9.1%)	0.94
VF or VT after the first day	1 (1.9 %)	0	1.00
AF	0	1 (4.5%)	0.29
First-degree AV block	0	1 (4.5%)	0.29
Heart failure	18 (34.6%)	2 (9.1%)	0.02
Death	3 ( 5.8%)	0 (0%)	0.55
MACE	27 (51.9%)	4 (18.2%)	0.01

^*^Number (percent). † Chi-square. MACE; was defined as the occurrence of death, VT/VF or heart failure.

## Conclusion

Studies have tried to explain complications in MI cases with LAD involvement, with respect to the involvement of conus branch. It is said that the presence of large conus branch may protect the right side of the interventricular septum and if a small conus branch is present, blood is supplied to both sides of the interventricular septum by LAD artery branches, thus the interventricular septum is left unprotected during LAD occlusion.­

## Competing of interests

The authors declare no conflict of interests**.**

## Ethical Approval

The study was approved under the code (94/3-9/6) in the Ethics council of Tabriz University of Medical Sciences.

## Acknowledgments

The authors would like to thank all patients participating in this study.
